# 2-Chloro­anilinium perchlorate

**DOI:** 10.1107/S1600536812023963

**Published:** 2012-06-16

**Authors:** Benhua Zhou, Jin Cai

**Affiliations:** a School of Chemistry and Chemical Engineering, Southeast University, Nanjing 210096, People’s Republic of China

## Abstract

In the crystal of the title compound, C_6_H_7_ClN^+^·ClO_4_^−^, a layer-like structure parallel to the *bc* plane is formed through N—H⋯O hydrogen bonds between the cations and anions. These layers are connected by weak C—H⋯O inter­actions, forming a three-dimensional network.

## Related literature

For general background to ferroelectric organic frameworks, see: Gray *et al.* (2002 ▶); Fu *et al.* (2007 ▶); Ye *et al.* (2009 ▶). For phase transitions of ferroelectric materials, see: Ye *et al.* (2006 ▶); Zhang *et al.* (2008 ▶); Zhao *et al.* (2008 ▶). For related structures, see: Gray *et al.* (2002 ▶); Balamurugan *et al.* (2010 ▶).
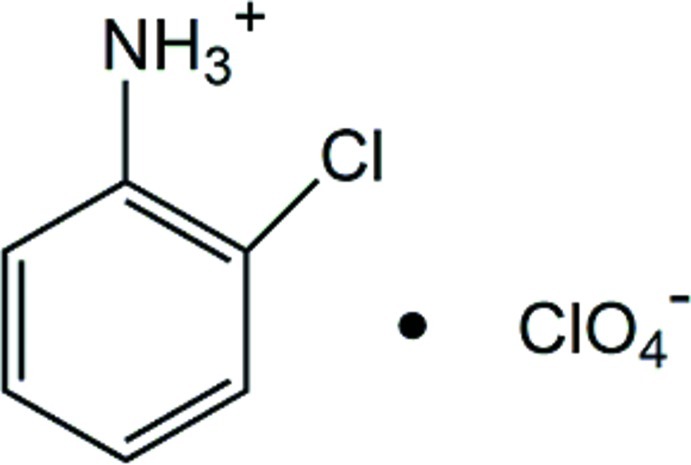


## Experimental

### 

#### Crystal data

C_6_H_7_ClN^+^·ClO_4_^−^
*M* *_r_* = 228.03
Monoclinic, 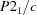

*a* = 11.069 (2) Å
*b* = 7.3093 (15) Å
*c* = 13.718 (5) Å
β = 125.737 (19)°
*V* = 900.9 (4) Å^3^
*Z* = 4
Mo *K*α radiation
μ = 0.70 mm^−1^
*T* = 293 K
0.20 × 0.20 × 0.20 mm

#### Data collection

Rigaku SCXmini diffractometer
Absorption correction: multi-scan (*CrystalClear*; Rigaku, 2005 ▶) *T*_min_ = 0.869, *T*_max_ = 0.869
8912 measured reflections
2060 independent reflections
1749 reflections with *I* > 2σ(*I*)
*R*_int_ = 0.040

#### Refinement

*R*[*F* ^2^ > 2σ(*F* ^2^)] = 0.046
*wR*(*F* ^2^) = 0.089
*S* = 2.26
2060 reflections
119 parameters
H-atom parameters constrained
Δρ_max_ = 0.31 e Å^−3^
Δρ_min_ = −0.38 e Å^−3^

### 

Data collection: *CrystalClear* (Rigaku, 2005 ▶); cell refinement: *CrystalClear*; data reduction: *CrystalClear*; program(s) used to solve structure: *SHELXS97* (Sheldrick, 2008 ▶); program(s) used to refine structure: *SHELXL97* (Sheldrick, 2008 ▶); molecular graphics: *DIAMOND* (Brandenburg & Putz, 2005 ▶); software used to prepare material for publication: *SHELXL97*.

## Supplementary Material

Crystal structure: contains datablock(s) I, New_Global_Publ_Block. DOI: 10.1107/S1600536812023963/zq2165sup1.cif

Structure factors: contains datablock(s) I. DOI: 10.1107/S1600536812023963/zq2165Isup2.hkl

Supplementary material file. DOI: 10.1107/S1600536812023963/zq2165Isup3.cml

Additional supplementary materials:  crystallographic information; 3D view; checkCIF report

## Figures and Tables

**Table 1:** Hydrogen-bond geometry (Å, °)

*D*—H⋯*A*	*D*—H	H⋯*A*	*D*⋯*A*	*D*—H⋯*A*
N1—H1*A*⋯O2^i^	0.89	2.21	2.978 (2)	145
N1—H1*A*⋯O4	0.89	2.63	3.333 (3)	136
N1—H1*B*⋯O3^ii^	0.89	2.04	2.911 (2)	168
N1—H1*C*⋯O4^iii^	0.89	2.29	3.022 (2)	140
N1—H1*C*⋯O2	0.89	2.51	3.039 (3)	118
C3—H3⋯O1^iv^	0.93	2.70	3.331 (3)	126

Symmetry codes: (i) 
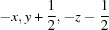
; (ii) 
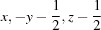
; (iii) 
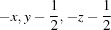
; (iv) 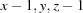.

**Fig. 1.**
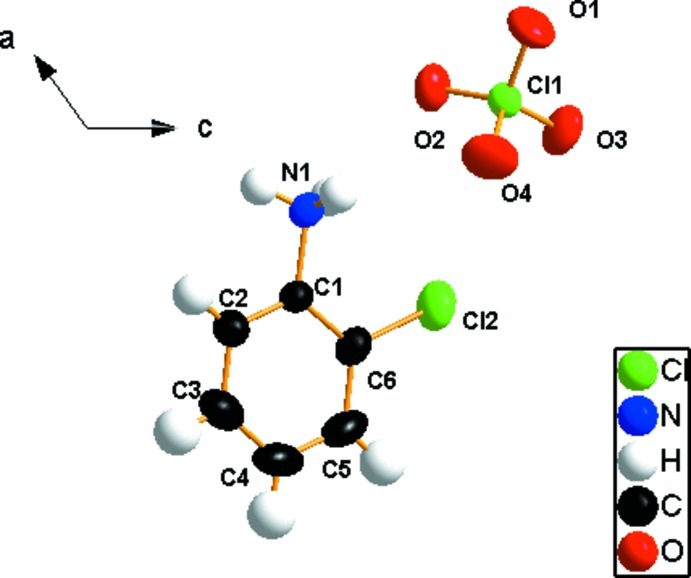
The molecular structure of the title compound, with the atomic numbering scheme and displacement ellipsoids drawn at the 30% probability level.

**Fig. 2.**
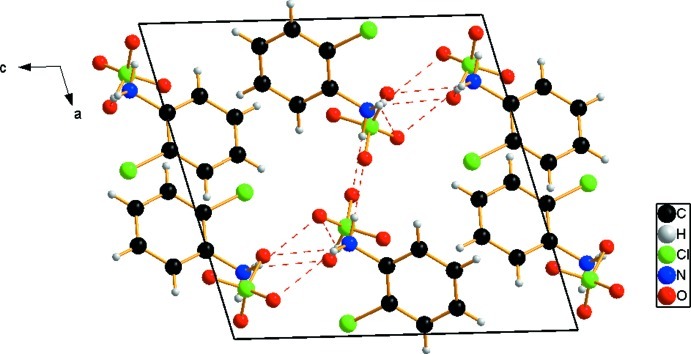
A view of the packing of the title compound, stacking along the b axis. Dashed lines indicate hydrogen bonds.

## supplementary crystallographic information

### Comment

The study of ferroelectric materials has received much attention and some materials have predominantly dielectric–ferroelectric performance (Ye *et al.*, 2006; Fu *et al.*, 2007; Zhao *et al.* 2008; Zhang *et al.*, 2008; Ye *et al.*, 2009). As a part of our work to obtain potential ferroelectric phase-transition materials, we report herein the crystal structure of title compound. Unluckily, the title compound exhibited no dielectric anomalies in the temperature range 93 – 453 K, suggesting that it might be only a paraelectric material.

The title compound, C_6_H_7_ClN^+^.ClO_4_^-^_,_ exhibits a two-dimensional layer-like structure parallel to the *bc* plane through intermolecular N—H···O hydrogen bonds between cations and anions (Fig. 1 & 2). Furthermore, the crystal structure is stabilized by weak C—H···O interactions which connect the two-dimensional layers.

The cation, C_6_H_7_ClN^+^, is reported in the literature with different counter-ions (Gray *et al.*, 2002; Balamurugan *et al.*, 2010).

### Experimental

For the preparation of the title compound, a water solution of perchloric acid (1 g) was added to the ethanol solution of 2-chlorobenzenamine. The resulting precipitate was filtered. Colorless crystals suitable for X-ray analysis were formed after several weeks by slow evaporation of the solvent at room temperature.

### Refinement

All H atoms were calculated geometrically and allowed to ride on the parent atom with C–H = 0.93 Å and *U*_iso_(H) = 1.2*U*_eq_(C), and with N–H = 0.89 Å *U*_iso_(H) = 1.2*U*_eq_(N).

### Crystal data

**Table:**

C_6_H_7_ClN^+^·ClO_4_^−^	*F*(000) = 464
*M**_r_* = 228.03	*D*_x_ = 1.681 Mg m^−^^3^
Monoclinic, *P*2_1_/*c*	Mo *K*α radiation, λ = 0.71073 Å
Hall symbol: -P 2ybc	Cell parameters from 2060 reflections
*a* = 11.069 (2) Å	θ = 3.3–27.5°
*b* = 7.3093 (15) Å	µ = 0.70 mm^−^^1^
*c* = 13.718 (5) Å	*T* = 293 K
β = 125.737 (19)°	Prism, colourless
*V* = 900.9 (4) Å^3^	0.20 × 0.20 × 0.20 mm
*Z* = 4	

### Data collection

**Table:**

Rigaku SCXmini diffractometer	2060 independent reflections
Radiation source: fine-focus sealed tube	1749 reflections with *I* > 2σ(*I*)
Graphite monochromator	*R*_int_ = 0.040
Detector resolution: 13.6612 pixels mm^-1^	θ_max_ = 27.5°, θ_min_ = 3.3°
CCD_Profile_fitting scans	*h* = −14→14
Absorption correction: multi-scan (*CrystalClear*; Rigaku, 2005)	*k* = −9→9
*T*_min_ = 0.869, *T*_max_ = 0.869	*l* = −17→17
8912 measured reflections	

### Refinement

**Table:**

Refinement on *F*^2^	Secondary atom site location: difference Fourier map
Least-squares matrix: full	Hydrogen site location: inferred from neighbouring sites
*R*[*F*^2^ > 2σ(*F*^2^)] = 0.046	H-atom parameters constrained
*wR*(*F*^2^) = 0.089	*w* = 1/[σ^2^(*F*_o_^2^) + (0.010*P*)^2^] where *P* = (*F*_o_^2^ + 2*F*_c_^2^)/3
*S* = 2.26	(Δ/σ)_max_ < 0.001
2060 reflections	Δρ_max_ = 0.31 e Å^−^^3^
119 parameters	Δρ_min_ = −0.38 e Å^−^^3^
0 restraints	Extinction correction: *SHELXL*, Fc^*^=kFc[1+0.001xFc^2^λ^3^/sin(2θ)]^-1/4^
Primary atom site location: structure-invariant direct methods	Extinction coefficient: 0.231 (7)

### Special details

**Table:**

Geometry. All e.s.d.'s (except the e.s.d. in the dihedral angle between two l.s. planes) are estimated using the full covariance matrix. The cell e.s.d.'s are taken into account individually in the estimation of e.s.d.'s in distances, angles and torsion angles; correlations between e.s.d.'s in cell parameters are only used when they are defined by crystal symmetry. An approximate (isotropic) treatment of cell e.s.d.'s is used for estimating e.s.d.'s involving l.s. planes.
Refinement. Refinement of *F*^2^ against ALL reflections. The weighted *R*-factor *wR* and goodness of fit *S* are based on *F*^2^, conventional *R*-factors *R* are based on *F*, with *F* set to zero for negative *F*^2^. The threshold expression of *F*^2^ > σ(*F*^2^) is used only for calculating *R*-factors(gt) *etc*. and is not relevant to the choice of reflections for refinement. *R*-factors based on *F*^2^ are statistically about twice as large as those based on *F*, and *R*- factors based on ALL data will be even larger.

### Fractional atomic coordinates and isotropic or equivalent isotropic displacement parameters (Å^2^)

**Table:**

	*x*	*y*	*z*	*U*_iso_*/*U*_eq_	
Cl2	−0.30288 (8)	−0.34063 (9)	−0.34203 (6)	0.0620 (3)	
N1	−0.12505 (19)	−0.1679 (2)	−0.41599 (16)	0.0359 (5)	
H1A	−0.0953	−0.0767	−0.3637	0.043*	
H1B	−0.0808	−0.1583	−0.4530	0.043*	
H1C	−0.1008	−0.2743	−0.3773	0.043*	
C1	−0.2866 (2)	−0.1583 (3)	−0.50492 (19)	0.0316 (5)	
C2	−0.3452 (3)	−0.0700 (3)	−0.6122 (2)	0.0421 (6)	
H2	−0.2824	−0.0175	−0.6287	0.051*	
C3	−0.4974 (3)	−0.0596 (3)	−0.6954 (2)	0.0556 (7)	
H3	−0.5375	−0.0005	−0.7683	0.067*	
C4	−0.5902 (3)	−0.1370 (3)	−0.6702 (3)	0.0584 (8)	
H4	−0.6928	−0.1305	−0.7267	0.070*	
C5	−0.5324 (3)	−0.2237 (3)	−0.5624 (3)	0.0536 (7)	
H5	−0.5954	−0.2742	−0.5455	0.064*	
C6	−0.3789 (3)	−0.2350 (3)	−0.4790 (2)	0.0391 (5)	
Cl1	0.07943 (6)	−0.17797 (7)	−0.07815 (5)	0.0349 (2)	
O1	0.21439 (19)	−0.0998 (3)	0.01753 (15)	0.0676 (6)	
O2	0.10292 (19)	−0.2780 (2)	−0.15577 (14)	0.0545 (5)	
O3	0.0235 (2)	−0.3048 (2)	−0.03403 (16)	0.0582 (5)	
O4	−0.0262 (2)	−0.0368 (2)	−0.14591 (16)	0.0658 (5)	

### Atomic displacement parameters (Å^2^)

**Table:**

	*U*^11^	*U*^22^	*U*^33^	*U*^12^	*U*^13^	*U*^23^
Cl2	0.0771 (6)	0.0615 (5)	0.0639 (5)	−0.0132 (3)	0.0506 (5)	0.0054 (3)
N1	0.0367 (11)	0.0319 (10)	0.0425 (11)	−0.0002 (8)	0.0251 (10)	0.0026 (8)
C1	0.0303 (12)	0.0283 (12)	0.0360 (12)	−0.0023 (8)	0.0192 (11)	−0.0060 (9)
C2	0.0420 (14)	0.0435 (14)	0.0394 (13)	−0.0055 (10)	0.0229 (12)	−0.0035 (10)
C3	0.0513 (17)	0.0539 (17)	0.0420 (14)	0.0032 (12)	0.0163 (14)	−0.0065 (12)
C4	0.0341 (15)	0.0604 (18)	0.0604 (19)	−0.0024 (12)	0.0161 (14)	−0.0211 (14)
C5	0.0423 (16)	0.0518 (16)	0.0750 (19)	−0.0159 (12)	0.0389 (15)	−0.0235 (14)
C6	0.0457 (15)	0.0315 (12)	0.0499 (14)	−0.0057 (10)	0.0335 (13)	−0.0077 (10)
Cl1	0.0385 (3)	0.0308 (3)	0.0353 (3)	−0.0012 (2)	0.0214 (3)	0.0015 (2)
O1	0.0539 (12)	0.0806 (13)	0.0458 (10)	−0.0245 (10)	0.0163 (10)	−0.0160 (9)
O2	0.0699 (13)	0.0548 (11)	0.0535 (11)	0.0007 (9)	0.0443 (11)	−0.0075 (8)
O3	0.0762 (13)	0.0476 (11)	0.0782 (13)	−0.0014 (8)	0.0606 (12)	0.0120 (9)
O4	0.0618 (12)	0.0375 (10)	0.0780 (13)	0.0180 (8)	0.0294 (11)	0.0155 (9)

### Geometric parameters (Å, º)

**Table:**

Cl2—C6	1.729 (2)	C3—H3	0.9300
N1—C1	1.464 (3)	C4—C5	1.376 (4)
N1—H1A	0.8900	C4—H4	0.9300
N1—H1B	0.8899	C5—C6	1.390 (3)
N1—H1C	0.8900	C5—H5	0.9300
C1—C2	1.374 (3)	Cl1—O1	1.4107 (17)
C1—C6	1.381 (3)	Cl1—O4	1.4237 (16)
C2—C3	1.379 (3)	Cl1—O3	1.4309 (15)
C2—H2	0.9300	Cl1—O2	1.4350 (16)
C3—C4	1.382 (4)		
			
C1—N1—H1A	109.4	C5—C4—C3	120.7 (2)
C1—N1—H1B	109.4	C5—C4—H4	119.6
H1A—N1—H1B	109.5	C3—C4—H4	119.6
C1—N1—H1C	109.6	C4—C5—C6	119.3 (2)
H1A—N1—H1C	109.5	C4—C5—H5	120.4
H1B—N1—H1C	109.5	C6—C5—H5	120.4
C2—C1—C6	120.6 (2)	C1—C6—C5	119.8 (2)
C2—C1—N1	119.84 (19)	C1—C6—Cl2	119.82 (18)
C6—C1—N1	119.5 (2)	C5—C6—Cl2	120.40 (19)
C1—C2—C3	119.8 (2)	O1—Cl1—O4	109.51 (12)
C1—C2—H2	120.1	O1—Cl1—O3	110.70 (11)
C3—C2—H2	120.1	O4—Cl1—O3	110.62 (11)
C2—C3—C4	119.9 (2)	O1—Cl1—O2	110.14 (11)
C2—C3—H3	120.1	O4—Cl1—O2	108.70 (11)
C4—C3—H3	120.1	O3—Cl1—O2	107.13 (11)
			
C6—C1—C2—C3	−0.7 (3)	N1—C1—C6—C5	179.00 (19)
N1—C1—C2—C3	−179.39 (19)	C2—C1—C6—Cl2	−178.46 (16)
C1—C2—C3—C4	0.3 (3)	N1—C1—C6—Cl2	0.2 (3)
C2—C3—C4—C5	0.4 (4)	C4—C5—C6—C1	0.5 (3)
C3—C4—C5—C6	−0.8 (4)	C4—C5—C6—Cl2	179.23 (18)
C2—C1—C6—C5	0.3 (3)		

### Hydrogen-bond geometry (Å, º)

**Table:**

*D*—H···*A*	*D*—H	H···*A*	*D*···*A*	*D*—H···*A*
N1—H1*A*···O2^i^	0.89	2.21	2.978 (2)	145
N1—H1*A*···O4	0.89	2.63	3.333 (3)	136
N1—H1*B*···O3^ii^	0.89	2.04	2.911 (2)	168
N1—H1*C*···O4^iii^	0.89	2.29	3.022 (2)	140
N1—H1*C*···O2	0.89	2.51	3.039 (3)	118
C3—H3···O1^iv^	0.93	2.70	3.331 (3)	126

Symmetry codes: (i) −*x*, *y*+1/2, −*z*−1/2; (ii) *x*, −*y*−1/2, *z*−1/2; (iii) −*x*, *y*−1/2, −*z*−1/2; (iv) *x*−1, *y*, *z*−1.
